# *Salmonella* serotypes in wild boars (*Sus scrofa*) hunted in northern Italy

**DOI:** 10.1186/1751-0147-55-42

**Published:** 2013-05-21

**Authors:** Mario Chiari, Mariagrazia Zanoni, Silvia Tagliabue, Antonio Lavazza, Loris G Alborali

**Affiliations:** 1 Central Diagnostic Laboratory, Istituto Zooprofilattico Sperimentale della Lombardia e dell’Emilia Romagna [IZSLER], “Bruno Ubertini”, Via Bianchi 7/9, Brescia 25124, Italy

**Keywords:** Italy, *Salmonella*, Wild boar

## Abstract

**Background:**

*Salmonella* species (spp.) are zoonotic enteric bacteria able to infect humans, livestock and wildlife.

However, little is known about the prevalence and the presence of the different serovars in wildlife. Considering the wide distribution of wild boars and the feeding behaviour (omnivorous scavengers), wild boars may be a good indicator for environmental presence of *Salmonella* spp. The aims of this study were to determine the presence of *Salmonella* spp. in hunted wild boars and to determine the serotype the isolated strains.

**Findings:**

Over three hunting seasons, the intestinal contents of 1,313 boars hunted in northern Italy were sampled and cultured. *Salmonella* spp. were isolated from 326 boars (24.82%). Thirty different serovars belonging to three different *S. enterica* spp. were found. Twenty-one serovars of *S. enterica* subsp. *Enterica* were found including the human pathogens *S.* Typhimurium and *S.* Enteritidis. In addition, nine serovars belonging to *S.enterica* subsp. *diarizonae* and *S. enterica* subsp. *houtenae* were detected.

**Conclusions:**

Considering the widespread occurrence of wild boars in Europe, the epidemiological role of this species in relation to salmonellosis might be relevant and should be further investigated. Wild boars may act as healthy carriers of a wide range of *Salmonella* serotypes.

## 

Salmonellosis was the second most common reported cause of zoonotic diseases in humans in Europe in 2010 where 99,020 cases were confirmed. Also, salmonellosis causes significant economic losses to the livestock industry [1,2]. Indeed, *Salmonella* species (spp.) are able to infect a wide range of domestic and wild animal species and have been isolated from the intestinal content of birds and mammals [3-5] including wildlife such as white-tailed deer [6], rabbits and wild boars (*Sus scrofa*) [7].

The occurrence of wild boar populations in most of Europe, including Italy, has required increased hunting to limit their expansion. It has been suggested, that wild boars might represent an important reservoir for zoonotic pathogens, including *Salmonella* spp. [8], and thus may act as a source of food borne infections in humans. Moreover, wild boars are omnivorous scavengers and they forage on almost anything including carrion, insects, and reptiles. Bacterial pathogens ingested by this behaviour may colonize the intestinal tract and especially in areas with intensive animal farming, wild boars are likely to be exposed to pathogens of farm animal origin, such as *S.* Enteritidis and *S.* Typhimurium. In contrast to the abundance of literature on the prevalence of *Salmonella* spp. in humans and in domestic pigs, little is known about the occurrence of this pathogen in wild boars [8]. In particular, limited data is available on the presence of the different serovars of *Salmonella* spp. Therefore, the aims of this study were to determine the prevalence of *Salmonella* spp. in wild boars hunted in northern Italy and to serotype the isolated strains.

Thanks to a health monitoring plan of free-ranging animals established in the Province of Brescia, Lombardy in northern Italy from 2007 to 2010, a total of 1,313 wild boars were sampled. In the three hunting season, 2007–2008, 2008–2009 and 2009–2010, the numbers of samples collected were 443, 442 and 428, respectively. During evisceration of killed boars, the large intestines were collected by hunters and delivered daily to the laboratory. For each animal the sex and age were registered. The age of the animals was determined on the basis of tooth eruption pattern [9]: individuals were considered “young” when <12 months of age, “sub-adults” when 13–24 months of age and “adults” when >24 months of age. The statistical analysis was performed by Yates’ chi-square test for comparisons of sex and age classes, using R 2.11.0 software [10].

The isolation method [11] was based on the pre-enrichment phase where 25 g of intestinal contents were inoculated into sterile sampling bags with 225 ml of buffered peptone water and incubated at 37°C for 24 hours; thereafter, 0.1 ml was inoculated on Modified Semisolid Rappaport Vassiliadis (MSRV; Oxoid, Hampshire, UK). Following incubation for 48 h at 41.5°C, *Salmonella* suspected colonies on MSRV plates were plated on two selective solid media: Xylose Lysine Deoxycholate agar (XLD; bioMérieux, Bagno a Ripoli, IT) and Brilliant Green Agar (BGA; Vacutest Kima, Arzergrande, IT). All presumptive *Salmonella* spp. isolates that developed colonies on XLD or on BGA were confirmed using biochemical tests (BBL™ Enterotube™ II, Becton Dickinson, Heidelberg, DE).

*Salmonella* spp. were isolated from 326 wild boar intestines (24.82%). In the hunting seasons of 2007/08, 2008/09 and 2009/10, 124 (27.99%), 80 (18.05%) and 122 (28.50%) samples tested positive, respectively (Table 1). This average high prevalence of positive animals (24.83%) was unexpected considering that salmonellosis in the European population of wild boars is considered uncommon [4]. A similar prevalence (22.1%) was found only recently in Portugal by analysing 77 wild boars, but just two serotypes (*S.* Typhimurium and *S.* Rissen) were identified [7]. *Salmonella* spp. has also been isolated in Switzerland from the tonsils and faeces of wild boars with a prevalence of 5.5%, and three serovars were identified [8].

**Table 1:** **Distribution of wild boars ( *Sus scrofa *) that tested positive for *Salmonella *spp., as classified by age, over three hunting seasons in northern Italy**

**Age**	**Hunting season**			**Total**
	**2007/08**	**2008/09**	**2009/10**	
Class 0 (<1 year old)	55	22	49	126
	*(44.35%)*	*(27.50%)*	*(40.16%)*	*(100%)*
Class 1 (<1-2 year old>)	33	24	31	88
	*(26.61%)*	*(30.00%)*	*(25.40%)*	*(100%)*
Class 2 (>2 year old)	22	25	33	70
	*(17.74%)*	*(18.75%)*	*(27.19%)*	*(100%)*
Undetermined	14	19	9	42
	*(11.30%)*	*(23.75%)*	*(7.25%)*	*(100%)*
Total	124	80	122	326
	(*100%)*	(*100%)*	(*100%)*	(*100%)*

Out of 326 positive animals, 152 (46.62%) were males and 158 (48.46%), were females while the sex was not recorded for 16 (4.92 %) animals. Prevalence did not differ between males and females (*χ*^2^= 1.40, df=1, *P* = 0.23). However, young animals (yearlings) seem to be more susceptible to intestinal colonization by *Salmonella* spp. than older animals (*χ*^2^= 79.53, df = 2, *P* < 0.001) (Table 1).

*Salmonella* spp. isolations were never associated with presence of macroscopic lesions during necropsy. This is different from observations of farmed wild boars where some serotypes of *Salmonella* such as *S.* Choleraesuis and *S.* Saintpaul are known to cause multifocal to diffuse fibrinonecrotic typhlocolitis [12,13].

Complete serological characterization of the 326 *Salmonella* strains was performed. This included rapid slide agglutination test for the detection of somatic antigens (Statens Serum Institut, Copenhagen, Denmark) and the hot tube agglutination test (Becton Dickinson, Heidelberg, Germany) according to Morris *et al.*[14] for the determination of flagellar antigen. The results of the antigen determination were used for the final serological characterization using the White -Kauffmann - Le Minos scheme [15]. The 326 *Salmonella* isolates belonged to 30 different serovars classified into three different subspecies of *S. enterica*: *S*. *enterica* subsp. *enterica* (n=259 strains, 79.45%, 21 serovars)*, S. enterica* subsp. *diarizonae* (n=38, 11.66%, 5 serovars) and *S. enterica* subsp. *houtenae* (n=29, 8.90%, 4 serovars) (Table 2).

**Table 2:** **Typing of *Salmonella *isolates from wild boars ( *Sus scrofa *) over three hunting seasons in northern Italy**

***S. enterica *serovars**	**Hunting season**			
	**2007/08**	**2008/09**	**2009/10**	**Total**
*S. enterica* subsp*. enterica*	118	62	79	259 *(79,45%)*
*Salmonella* Typhimurium	26	4	2	32 *(9.82%)*
*Salmonella* Ball	14	8	3	25 *(7.67%)*
*Salmonella* Choleraesuis	0	0	1	1 *(0.31%)*
*Salmonella* Coeln	36	13	22	71 *(21.78%)*
*Salmonella* Derby	5	2	1	8 *(2.45%)*
*Salmonella* Enterica 4,5,12:i:-	0	9	4	13 *(3.99%)*
*Salmonella* Enteritidis	3	7	6	16 *(4.91%)*
*Salmonella* Ferruch	2	0	0	2 *(0.61%)*
*Salmonella* Kottbus	4	1	0	5 *(1.53%)*
*Salmonella* Infantis	0	0	3	3 *(0.92%)*
*Salmonella* Livingstone	1	0	0	1 *(0.31%)*
*Salmonella* Llobregat	0	0	1	1 *(0.31%)*
*Salmonella* Manhattan	0	0	4	4 *(1.23%)*
*Salmonella* Michigan	0	3	0	3 *(0.92%)*
*Salmonella* Muenster	0	2	0	2 *(0.61%)*
*Salmonella* Munchen	0	0	3	3 *(0.92%)*
*Salmonella* Napoli	5	2	12	19 *(5.83%)*
*Salmonella* Newport	0	1	0	1 *(0.31%)*
*Salmonella* Saintpaul	0	4	0	4 *(1.23%)*
*Salmonella* Thompson	12	4	7	23 *(7.06%)*
*Salmonella* Veneziana	10	2	10	22 *(6.75%)*
*S. enterica* subsp. *diarizonae* (5 serovars)	2	9	27	38 *(11.66%)*
S*. enterica* subsp. *houtenae* (4 serovars)	4	9	16	29 *(8.90%)*
Total	124	80	122	326 *(100%)*

According to the nomenclature [15], the *Salmonella enterica* cluster is divided into six subspecies each containing a variable number of serovars. In this study, three subspecies were isolated. *S. enterica* subsp. *enterica* is considered the most widespread and includes many serovars, usually found in mammals, food and also in the environment [2]. A total of 259 isolates of *S. enterica* subsp*. enterica* belonging to 21 different serovars (Table 2) were identified during the three hunting seasons. In particular, 11 serovars were detected in just one season, two serovars in two seasons and eight in all three seasons. Such high variability might indicate different sources of exposure from activities such as livestock farming and waste disposal, or exposure from other wild species such as birds and amphibians [16]. *S.* Typhimurium was identified in all three seasons of this study. *S.* Typhimurium was the second most frequently identified *S. enterica* subsp. *enterica* serovar in European human salmonellosis cases reported in 2010 [2], and it deserves special attention due to its virulence and high prevalence of antibiotic resistance [17]. Similar importance should also be given to both *S.* Enteritidis, which was the most frequently identified serovar in European human salmonellosis cases in 2010 and to *S.* Derby, which was included in the top 10 serovars reported in human cases in 2010 in Europe [2]. The isolation of a relatively high number of *S. enterica* subsp. *enterica* serovars and their relative frequencies (*S.* Coeln 21.78%, *S.* Ball 7.67%, *S.*Thompson 7.06%, *S.* Veneziana 6.75% and *S.* Napoli 5.83%) prove how wild boars can consistently be indicators of the presence of a great variety of serotypes in the environment. It should be underlined that information in the literature on the identification, distribution and pathogenic role of *S.* Coeln is very scarce, though this serotype was considered the cause of an outbreak in humans in France [18]. In addition, the isolation of *S.* Napoli and of *S.* Enterica 4,5,12:i:-, which is a monophasic variant of *S.* Typhimurium, might contribute to the understanding of the epidemiology and distribution of these serotypes, which are considered emerging serovars both in humans and animals [19,20]. Further molecular investigation and the use of a more sensitive method such as pulsed-field gel electrophoresis (PFGE) [21] for fingerprinting *Salmonella* serovars, would be necessary to correctly classify the various strains, but it is beyond the aim of this study.

Other isolates from wild boars belonged to *S. enterica* subsp. *diarizonae* and *S. enterica* subsp. *houtenae*. According to the Enternet network report such serovars were not detected during official livestock surveys carried out in the same area over the same time period (data not published).

The risks factors for human salmonellosis are numerous: infection may indirectly arise in agricultural areas from the contamination of vegetable products, through direct animal contact, during hunting and carcass manipulation, or directly from ingestion of contaminated meat or meat products [16]. The contamination of wild boar carcasses by intestinal contents containing enteric pathogenic bacteria could not be excluded in hunted animals.

The results are in agreement with the hypothesis that, although domestic animals are the major reservoir of *Salmonella* spp., the wild boars omnivorous feeding habits makes this animal more exposed to intestinal contamination and possible colonization by *Salmonella* spp. In addition, the potential role of wild boars as carriers and faecal spreaders of *Salmonella* spp. in the natural epidemiological cycle of this enteric pathogen must be highlighted. In this sense wild boars can act as a good sentinel species for the presence of environmental *Salmonella* serotypes, some of which have zoonotic implications.

## Competing interests

The authors declare no competing interests.

## Authors’ contributions

MC designed the study, performed the isolation of *Salmonella* spp. and drafted the first version of the manuscript. MZ initiated the study and supervised the isolation of *Salmonella* spp. and the statistical analysis. ST performed the serological characterization of the isolates. AL and LA supervised in the design of the study and the epidemiological analysis. All authors have read and accepted the final manuscript.

## References

[B1] Gortàzar C Ferroglio E Hofle U Frolich K Vincente J Diseases shared between wildlife and livestock: a European perspective Eur J Wildl Res 2007 53 241 256 10.1007/s10344-007-0098-y

[B2] European Food Safety Authority (EFSA) The community summary report on trends and sources of zoonoses, zoonotic agents, and food-borne outbreaks in the European union in 2010 E.F.S.A. Journal 2012 10 2597

[B3] Refsum T Handeland K Baggesen DL *Salmonellae* in avian wildlife in Norway from 1969 to 2000 Appl Environ Microb 2002 68 5595 5599 10.1128/AEM.68.11.5595-5599.2002 PMC129881 12406754

[B4] Wahlström H Tysen E Olsson Engvall E Brändström B Eriksson E Mörner T Vågsholm I Survey of *campylobacter* species, VTEC O157 and *salmonella* species in Swedish wildlife Vet Rec 2003 153 74 80 10.1136/vr.153.3.74 12892266

[B5] Millán J Aduriz G Moreno B Juste RA Barral M *Salmonella* isolates from wild birds and mammals in the Basque country (Spain) Rev Sci Tech Off Int Epiz 2004 23 905 911 10.20506/rst.23.3.1529 15861885

[B6] Renter DG Gnad DP Sargeant JM Hygnstrom SE Prevalence and serovars of *salmonella* in feces of free-ranging white-tailed deer (*odocoileus virginianus)* in Nebraska J Wildl Dis 2006 42 699 703 17092906 10.7589/0090-3558-42.3.699

[B7] Vieira-Pinto M Morais L Caleja C Themudo P Torres C Igrejas G Poeta P Martins C *Salmonella* sp. In game (*Sus scrofa* and *oryctolagus cuniculus*) Foodborne Pathog Dis 2011 8 739 740 10.1089/fpd.2010.0742 21254910

[B8] Wacheck S Fredriksson-Ahomaa M König M Stolle A Stephan R Wild boars as an important reservoir for foodborne pathogens Foodborne Pathog Dis 2010 7 307 312 10.1089/fpd.2009.0367 19899962

[B9] Saez-Royuela C Gomariz C Telleria JL Age determination of European wild boar [*Sus scrofa*] Wildl Soc Bull 1989 17 326 329

[B10] R Development Core Team R: a language and environment for statistical computing 2010 Vienna: R Foundation for Statistical Computing

[B11] International Organization for Standardization (ISO) Microbiology of food and animal feeding stuff—horizontal method for the detection of salmonella. ISO 6579 2002 Geneva, Switzerland: International Organization for Standardization

[B12] Ecco R Guedes RMC Tury E Santos HL Jr Perecmanis S Outbreak of enterocolitic salmonellosis on a wild pig farm Vet Rec 2006 158 242 243 10.1136/vr.158.7.242 16489165

[B13] Perez J Astorga R Carrasco L Mendez A Perea A Sierra MA Outbreak of salmonellosis in farmed European wild boars [*Sus scrofa ferus*] Vet Rec 1999 145 464 465 10.1136/vr.145.16.464 10576283

[B14] Morris GK Steele CD Wells JG Evaluation of plastic multi-well plates for serological screening of *salmonella* cultures with Spicer-Edwards pooled Antisera Appl Microbiol 1972 24 846 848 4640740 10.1128/am.24.5.846-848.1972 PMC380677

[B15] Grimont PAD Weill FX Antigenic formulae of the *salmonella* serovars [WHO collaborating center for reference and research on salmonella 2007 9 Paris: Institut Pasteur http://www.pasteur.fr/ip/portal/action/WebdriveActionEvent/oid/01s-000036-089

[B16] Paulsen P Smulders FJM Hilbert F *Salmonella* in meat from hunted game: a central Europe perspective Food Res Int 2012 45 609 616 10.1016/j.foodres.2011.06.055

[B17] Cruchaga S Echeita A Aladueña A García-Peña J Frias N Usera MA Antimicrobial resistance in salmonellae from humans, food and animals in Spain in 1998 J Antimicrob Chemother 2001 47 315 321 10.1093/jac/47.3.315 11222564

[B18] Haeghebaert S Vaillant V Portal H Bouvet P Minet JC Grimont F Epidemic of salmonellosis due to *salmonella enterica* serotype coeln. France, November 1998 Bulletin Épidémiologique Hebdomadaire 2000 36 151 153

[B19] Graziani C Busani L Dionisi AM Caprioli A Ivarsson S Hedenström I Luzzi I Virulotyping of *salmonella enterica* serovar Napoli strains isolated in Italy from human and nonhuman sources Foodborne Pathog Dis 2011 8 997 1003 10.1089/fpd.2010.0833 21561382

[B20] Switt AI Soyer Y Warnick LD Wiedmann M Emergence, distribution, and molecular and phenotypic characteristics of salmonella enterica serotype 4,5,12:i:-Foodborne Pathog Dis 2009 6 407 415 10.1089/fpd.2008.0213 19292687 PMC3186709

[B21] Murase T Okitsu T Suzuki R Morozumi H Matsushima A Nakamura A Yamai S Evaluation of DNA fingerprinting by PFGE as an epidemiologic tool for salmonella infections Microbiol Immunol 1995 39 673 676 8577280 10.1111/j.1348-0421.1995.tb03255.x

